# Activated thrombin-activatable fibrinolysis inhibitor (TAFIa) attenuates breast cancer cell metastatic behaviors through inhibition of plasminogen activation and extracellular proteolysis

**DOI:** 10.1186/s12885-016-2359-1

**Published:** 2016-05-24

**Authors:** Zainab A. Bazzi, Danielle Lanoue, Mouhanned El-Youssef, Rocco Romagnuolo, Janice Tubman, Dora Cavallo-Medved, Lisa A. Porter, Michael B. Boffa

**Affiliations:** Department of Chemistry & Biochemistry, University of Windsor, Windsor, ON N9J 3P4 Canada; Department of Biological Sciences, University of Windsor, Windsor, ON N9J 3P4 Canada

**Keywords:** TAFI, Thrombomodulin, Breast cancer, Metastasis, Extracellular proteolysis, Plasminogen

## Abstract

**Background:**

Thrombin activatable fibrinolysis inhibitor (TAFI) is a plasma zymogen, which can be converted to activated TAFI (TAFIa) through proteolytic cleavage by thrombin, plasmin, and most effectively thrombin in complex with the endothelial cofactor thrombomodulin (TM). TAFIa is a carboxypeptidase that cleaves carboxyl terminal lysine and arginine residues from protein and peptide substrates, including plasminogen-binding sites on cell surface receptors. Carboxyl terminal lysine residues play a pivotal role in enhancing cell surface plasminogen activation to plasmin. Plasmin has many critical functions including cleaving components of the extracellular matrix (ECM), which enhances invasion and migration of cancer cells. We therefore hypothesized that TAFIa could act to attenuate metastasis.

**Methods:**

To assess the role of TAFIa in breast cancer metastasis, in vitro migration and invasion assays, live cell proteolysis and cell proliferation using MDA-MB-231 and SUM149 cells were carried out in the presence of a TAFIa inhibitor, recombinant TAFI variants, or soluble TM.

**Results:**

Inhibition of TAFIa with potato tuber carboxypeptidase inhibitor increased cell invasion, migration and proteolysis of both cell lines, whereas addition of TM resulted in a decrease in all these parameters. A stable variant of TAFIa, TAFIa-CIIYQ, showed enhanced inhibitory effects on cell invasion, migration and proteolysis. Furthermore, pericellular plasminogen activation was significantly decreased on the surface of MDA-MB-231 and SUM149 cells following treatment with various concentrations of TAFIa.

**Conclusions:**

Taken together, these results indicate a vital role for TAFIa in regulating pericellular plasminogen activation and ultimately ECM proteolysis in the breast cancer microenvironment. Enhancement of TAFI activation in this microenvironment may be a therapeutic strategy to inhibit invasion and prevent metastasis of breast cancer cells.

## Background

Metastasis is the process by which malignant cells migrate from the site of the primary tumor to distant parts of the body [1]. This phenomenon is responsible for the majority of breast cancer related deaths. The breast tumor microenvironment facilitates metastasis by providing the necessary factors (such as stromal cells, signaling molecules, and proteolytic enzymes) to initiate the metastatic process. Importantly, metastasis is mediated by the interaction between the tumor cells, stromal cells and the extracellular matrix (ECM) [2, 3]. Proteins in the tumor microenvironment influence the progression of cancer by providing a favourable environment for ECM degradation. Specifically, the presence of proteases such as plasmin and matrix metalloproteinases (MMPs) mediate extracellular proteolysis, resulting in ECM degradation [4, 5]. Degradation of the ECM promotes cell migration and invasion and also releases latent growth factors that stimulate cell proliferation [1].

The plasminogen activation system (PAS) plays a vital role in extracellular proteolysis. Plasminogen is a zymogen that is converted to its active form plasmin by urokinase plasminogen activator (uPA) and tissue-type plasminogen activator (tPA) [4], with uPA playing the more important role in pericellular plasminogen activation [6]. Plasmin is a serine protease of broad specificity that cleaves multiple substrates, including ECM proteins, latent growth factors and a number of pro-MMPs [4]. Plasmin therefore is considered a pro-metastasis enzyme [7]. The PAS is active in many types of cancers, including breast cancer [4]. Specifically, uPA and its receptor, urokinase plasminogen activator receptor (uPAR) are expressed in tumor cells, as well as tumor-associated stromal cells [4, 8]. Developing methods to target the PAS could be key in inhibiting cancer cell invasion and metastasis [9].

Thrombin activatable fibrinolysis inhibitor (TAFI), also known as carboxypeptidase B, U or R [10], is a plasma zymogen expressed mainly in the liver but also found in megakaryocytes (leading to a platelet pool of TAFI) and macrophages [11]. TAFI is converted to activated TAFI (TAFIa) by proteolytic cleavage at Arg92 either by thrombin, plasmin or thrombin in complex with the endothelial cofactor thrombomodulin (TM). TAFIa is a basic carboxypeptidase, which removes carboxyl-terminal arginine and lysine residues from peptide and protein substrates, including fibrin degradation products (FDPs), anaphylatoxins C3a and C5a, thrombin-cleaved osteopontin and cell-surface plasminogen receptors [12–15].

Intriguingly, TAFIa is intrinsically unstable, with a half-life of 8–15 min at 37°C. This instability is considered to be the physiological means of TAFIa inhibition, as no endogenous inhibitors exist [16]. Ceresa et al. characterized a mutant of TAFIa with a 180-fold enhancement in half-life compared to wildtype TAFIa [17]. This stable mutant of TAFIa contains five point mutations in the instability region, specifically S305C, T325I, T329I, H333Y, H335Q and is therefore named TAFI-CIIYQ.

The ability of TAFIa to cleave cell-surface plasminogen receptors raises the possibility that TAFIa could modulate the PAS in the tumor microenvironment, thereby attenuating the metastatic potential of the tumor cells. Furthermore, TM, the cofactor necessary for physiological activation of TAFI has been characterized as an anti-metastatic factor. In vitro studies have shown that TM can inhibit invasion of fibrosarcoma cells through activation of TAFI [18]. An important in vivo study demonstrated that the anti-metastatic potential of TM could be attributed to its thrombin-binding domain [19]. In addition, expression of TM in breast cancer tumors is inversely correlated to malignancy [20]. Therefore, we hypothesized that the anti-metastatic potential of TM may be mediated by its ability to enhance the activation of TAFI. Here, we address this hypothesis using an in vitro model of the breast tumor microenvironment.

## Methods

### Cell culture

MDA-MB-231 and HTB-126 cells (ATCC) were grown in Dulbecco’s modified eagle medium (DMEM/F12) (Invitrogen), containing 10 % fetal bovine serum (FBS) and 1 % antibiotic-antimycotic (10 units/mL penicillin G sodium, 10 μg/mL streptomycin sulfate and 25 ng/mL amphotericin B) (Invitrogen). SUM149 cells (a kind gift from Dr. Stephen Ethier, Karmanos Cancer Institute) were cultured in DMEM/F12 containing 5 % FBS, 1 % antibiotic-antimycotic, 10 μg/mL insulin (Sigma) and 0.5 μg/mL hydrocortisone (Sigma). MCF7 cells (ATCC) were grown in RPMI-1640 medium (Gibco) containing 10 % FBS and 1 % antibiotic-antimycotic. MCF10A (ATCC) and MCF10CA1a (obtained from the Karmanos Biobanking and Correlative Sciences Core) were maintained in DMEM-F12 media containing 0.5 μg/ml hydrocortisone, 10 μg/ml insulin, 20 ng/ml human EGF and 5 % (vol/vol) heat inactivated horse serum. Human embryonic kidney (HEK293) cells (ATCC) were cultured in minimum essential medium (MEM) (Gibco) supplemented with 5 % FBS and 1 % antibiotic-antimycotic. THP-1 acute monocytic leukemia cells (ATCC) were cultured in RPMI-1640 medium adjusted to contain 4.5 g/L glucose, 10 mM HEPES pH 7.4 and 1.0 mM sodium pyruvate, and supplemented with 10 % (v/v) fetal bovine serum (ATCC), 1 % (v/v) antibiotic-antimycotic, and 50 μM β-mercaptoethanol. THP-1 monocytes were differentiated by the addition of 100 nM phorbol 12-myristate 13-acetate to the medium for 72 h. Human umbilical vein endothelial cells (HUVECs) (Lonza) were grown in EGM Complete Medium (Lonza). All cells were sub-cultured in 100 mm cell culture plates and were kept in an incubator maintaining conditions of 37 **°**C, 5 % CO_2_.

### Cloning, expression and purification of recombinant TAFI

The full-length TAFI-CIIYQ cDNA sequence [17] present in the pNUT vector [21] was used as a template for PCR amplification. To insert TAFI-CIIYQ in the pcDNA4A vector, unique *Pst*I and *Age*I restriction sites were engineered at the 5′ and 3′ ends of the sequence through PCR. PCR amplification was performed with Phusion HF DNA polymerase (New England Biolabs) as per manufacturer’s protocol, using TAFI-CIIYQ-pNUT as the template (10 ng/μL) and the following primer pair: sense 5′-AAA CTG CAG TTG GGA TGA AGC TTT GC-3′ and anti-sense 5′-GGA CCG GTA ACA TTC CTA ATG ACA TGC CAA G-3′. Using *Pst*I and *Age*I, TAFI-CIIYQ cDNA was ligated into the pcDNA4A plasmid in-frame with a carboxyl-terminal His_6_-tag-encoding sequence. TAFI-WT [21] was cloned into pcDNA4A using the same method.

HEK293 cells were seeded into a 6-well plate at a density of 1.5 ×10^6^ cells per well. Cells were transfected using 1 μg of TAFI-pcDNA4A expression plasmids and Mega Tran 1.0 (OriGene) according to manufacturer’s protocol. Stably-expressing cell lines were selected by culturing in the presence of 100 μg/mL zeocin (Invitrogen). Stable cell lines were grown in roller bottle culture for the production of recombinant TAFI in OptiMEM (Gibco) supplemented with 1 % antibiotic/antimycotic.

Recombinant TAFI (rTAFI) was purified using a Ni^2+^ Sepharose column (GE Healthcare Life Sciences), utilizing the His_6_-tag at the carboxyl-terminal of the protein. Conditioned medium harvested from the cells was supplemented with 50 mM NaH_2_PO_4_, 0.5 M NaCl, 1 mM β-ME 10 % glycerol, pH 7.9 (buffer A), containing 5 mM imidazole. The column was washed and eluted with wash and elution buffers, which were buffer A containing 10 mM and 400 mM imidazole, respectively. Eluted rTAFI was dialyzed overnight at 4 °C against HBS (0.02 M HEPES pH 7.4, 0.15 M NaCl) containing 5 % glycerol. Dialyzed rTAFI was then concentrated by ultracentrifugation using Amicon Ultra Centrifugal Filters (Fisher Scientific) at 4 °C with a buffer exchange using HBS containing 0.01 % (v/v) Tween-80 (HBST).

### RNA extraction and quantitative real-time PCR

RNA was extracted from various cell lines using RNeasy Plus Mini Kit (Qiagen), as per the manufacturer’s protocol. Quantitative real-time PCR (qRT-PCR) was completed using iTaq one-step RT-PCR kit with SYBR® green (BioRad). The following primers were used for qRT-PCR: TM sense: 5′ CCC TGA ACA AGA ATT GGA AGC T 3′; TM antisense: 5′ GGA GCC TAG GAT TCT GCA TTT CTA 3′; TAFI sense: 5′ GCT GCC GGA GCG TTA CAT 3′; TAFI antisense: 5′ CAT TCC TAA TGA CAT GCC AAG CT 3′; GAPDH sense: 5′ GGA GCC AAA AGG GTC ATC ATC 3′; GAPDH antisense: 5′ GTT CAC ACC CAT GAC GAA CAT G 3′.

### Plasminogen activation assays

Plasminogen activation assays were performed as previously described by Romagnuolo et al. [22]. Briefly, MDA-MB-231 and SUM149 cells were grown to confluency in black, clear-bottom 96 well plates. Various TAFI concentrations were activated in Hank’s buffer saline solution (HBSS) (0.137 M NaCl, 5.4 mM KCl, 0.25 mM Na_2_HPO_4_, 0.44 mM KH_2_PO_4_, 1.3 mM CaCl_2_, 1 mM MgSO_4_, 4.2 mM NaHCO_3_, 1 g/L glucose and 0.1 % BSA) with 25 nM thrombin, 100 nM TM, and 5 mM CaCl_2_ for 10 min at room temperature. Following the 10-min incubation, thrombin was inhibited with PPAck. Cells were washed twice with HBSS. Activated TAFI (1–50 nM, final) was added to cells for 30 min at 37 **°**C. Cells were then washed twice with HBSS and a solution containing 300 nM plasminogen (purified from human plasma by lysine-sepharose affinity chromatography as previously described [23]), 10 pM uPA (Calbiochem) and 40 mM of the fluorogenic plasmin substrate H-D-Val-Leu-Lys-AMC (Bachem). Hydrolysis of H-D-Val-Leu-Lys-AMC was monitored for an hour at 37 **°**C at excitation and emission wavelengths of 370 and 470 nm, respectively.

### Cell invasion and migration assays

Cell culture inserts (BD Biosciences) with a pore size of 8 μm were coated with 2 mg/mL Cultrex reconstituted basement membrane extract (Trevigen) for 1 h at room temperature. MDA-MB-231 and SUM149 cells (1 × 10^5^) in serum-free medium were seeded into the upper chamber. Complete medium was added to the lower chamber with either 10 ug/mL potato tuber carboxypeptidase inhibitor (PTCI) (Sigma) or 10 nM rabbit TM (Haemotologic Technologies). Cells were allowed to invade for 24 h at 37 **°**C. Following 24 h, non-invaded cells in the upper chamber were removed. Invaded cells were fixed with cold methanol and stained with 0.25 % crystal violet. Images were taken of five fields of view with a 20× objective and cell numbers in each field were determined by counting.

For TAFIa treatment, recombinant TAFI was activated with 25 nM thrombin (Haematologic Technologies), 100 nM TM, and 5 mM CaCl_2_ for 10 min at room temperature. Thrombin was inhibited with 200 nM H-D-Phe-Pro-Arg chloromethylketone (PPAck; Calbiochem) and placed on wet ice. The activation mixture was supplemented with DMEM containing 10 % FBS and 1 % antibiotic and added to the lower chamber to achieve final TAFIa concentrations of 1, 5, 25, or 50 nM. Mock activations of rTAFI were carried out in the same manner but without thrombin.

Cell migration assays were performed using the same protocol as cell invasion assays, except that the cell culture inserts were not coated with Cultrex and the cells were seeded in the upper chamber at a lower density (3 × 10^4^ cells/well).

### Cell metabolism assay

MDA-MB-231 and SUM149 cells (2.5 × 10^3^/well) were seeded into a 96 well plate. Cells were grown for 24 h. PTCI or TM was then added to the cells in fresh medium for 24 h. Following the 24 h, WST-1 reagent (Roche) was added and incubated for 2 h at 37**°**C. The absorbance was then measured at 450 nm using a plate-reading spectrophotometer.

#### Live-cell proteolysis assay

Live-cell proteolysis experiments were carried out as described by Victor et al. [24]. Round glass-bottom dishes (MatTek) were coated with Cultrex containing 25 μg/mL dye quenched (DQ)-collagen IV (Invitrogen). MDA-MB-231 cells and SUM149 cells were labeled with CellTracker Orange (Invitrogen) in serum-free medium for 1 h. Cells were allowed to recover in complete medium before 2 × 10^4^ (SUM149) or 1 × 10^4^ cells (MDA-MB-231) were seeded onto the Cultrex-coated glass. Dishes were then incubated at 37 **°**C for 45 min. Following the incubation, complete growth medium containing 2 % Cultrex was added to the cells, with or without 10 μg/mL PTCI. Cells were incubated at 37 **°**C and 5 % CO_2_ for 48 h. Following incubation, cells were imaged using an Olympus IX81 confocal microscope.

For TAFIa treatment, activated (50 nM) and mock-activated (50 nM) rTAFI mixtures were prepared as described above. Glass-bottom dishes were prepared and cells were seeded as described above. Each plate was supplemented with 2 mL of DMEM medium containing 10 % FBS, 1 % antibiotic, 2 % Cultrex and the activation mixture with or without activated TAFI. Cells were incubated at 37**°**C and 5 % CO_2_ for 48 h.

Quantitative analysis of the confocal microscopy data was performed using the following protocol. Each of the images (15–20 depending on the size of the spheroid) of the z-stack in the experiment was initially in TIFF RGB format, which were then converted to 8-bit grayscale prior to processing. The images were then filtered to remove granulation using a median filter. The brightness curves were trimmed so that areas of high intensity did not discard other areas of interest when thresholding; the identical trimming method was conducted for each image. Each image was then filtered and tuned (again using the same parameters in each case), a threshold based on Otsu’s technique [25] was applied to the image which converts it to a binary format. The resultant image contains an accurate depiction of the shape and contour of the areas of interest. At this stage, the areas of each image were then calculated by matrix summation. The average intensity of an image was then determined by applying the binary image as a mask to the original grayscale image and calculating the mean pixel intensity of the resulting matrix. Each pixel is represented as an 8-bit grayscale value and therefore represented as a number between 0 and 255. This algorithm was applied to each image in the same exact manner without any modifications. The data were represented as a ratio of the intensity of the green fluorescence to the area of the cells, averaged over the z-stack.

### Statistical analysis

Data from the experiments were presented as means of at least 3 independent experiments ± standard error of the mean (SEM). Analysis of the data to identify statistically significant differences amongst experimental conditions was completed using one-way ANOVA with SPSS software (version 22); pairwise comparisons between conditions were performed using a Tukey post-hoc analysis. *p* values <0.05 were considered statistically significant.

## Results

### TAFI and TM are expressed in breast cancer cell lines

**Fig. 1:**
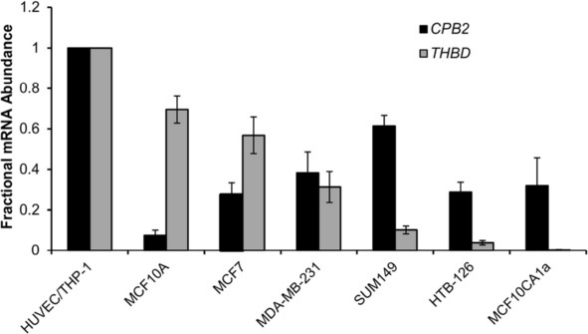
Expression of *CPB2* (TAFI) and *THBD* (thrombomodulin) mRNA in breast cancer cell lines. RNA was extracted from various breast cancer cell lines and expression of *CPB2* and *THBD* was analyzed using qRT-PCR. Expression of *CPB2* and *THBD* were normalized to *GAPDH* mRNA levels in all cells. The data are expressed relative to THP-1 macrophages (*CPB2*) or HUVECs (*THBD*) and are the means ± SEM of 3–4 independent experiments, and are arranged in decreasing order of *THBD* expression from left to right. *: *p* <0.05 versus MCF10A (*CPB2*) and †: *p* <0.05 versus MCF10A (*THBD*)

Expression of *CPB2* (the gene encoding TAFI) was assessed in various breast cancer cell lines, using qRT-PCR (Fig. 1). *CPB2* mRNA was detectable in all of the examined breast cancer cell lines, albeit at a lower level in all cases compared to the positive control THP-1 macrophages (Fig. 1), which is correspondingly much lower than reported in liver or a cultured hepatoma cell line [11]. *CPB2* mRNA levels in the highly malignant and invasive MDA-MB-231, HTB-126, and MCF10ACA1a cell lines were comparable to mRNA levels in the non-invasive [26] MCF7 cell line. Therefore, levels of *CPB2* mRNA do not appear to show any relationship to the malignancy of the breast cancer cell lines.

It has been reported that TM expression is inversed correlated with malignancy in prostate, breast and lung cancer [20, 27–29]. In contrast to *CPB2*, the expression levels of *THBD* (the gene encoding TM) were found to be generally inversely correlated to malignancy (Fig. 1). This relationship is revealed when the cell lines are arranged in decreasing order of *THBD* expression from left to right, as the more malignant cell lines are on the right.

### TAFIa inhibits plasminogen activation on both MDA-MB-231 and SUM149 cell lines

**Fig. 2:**
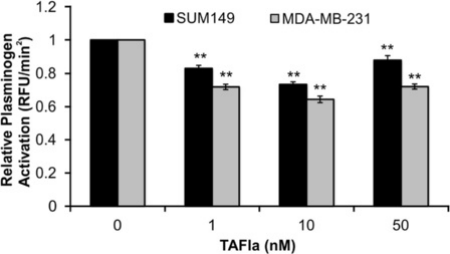
TAFIa inhibits pericellular plasminogen activation on breast cancer cell lines. SUM149 (*black bars*) and MDA-MB-231 cells (*grey bars*) were treated with varying concentrations of TAFIa (1–50 nM) for 30 min at 37 °C. Plasminogen activation assay was carried out with 300 nM plasminogen, 10 pM uPA and a fluorescent plasmin substrate. The rate of formation of plasmin (taken as the slope of the plot of change in fluorescence versus time squared) was monitored for 1 h at 37 °C. The data represent the means ± SEM of 4–5 independent experiments and are expressed relative to the control in the absence of TAFIa. **: *p* <0.01 relative to control

Addition of TAFIa resulted in a decrease in plasminogen activation of up to 30 % in both MDA-MB-231 and SUM149 cells (Fig. 2). This decrease, however, was not strictly dose-dependent, as the magnitude of the effect tended to decrease at the highest concentrations of TAFIa. The ability of TAFIa to decrease cell surface plasminogen activation is consistent with its ability to decrease extracellular collagen proteolysis.

### TAFIa directly inhibits cell invasion and migration of MDA-MB-231 and SUM149 cell lines

**Fig. 3:**
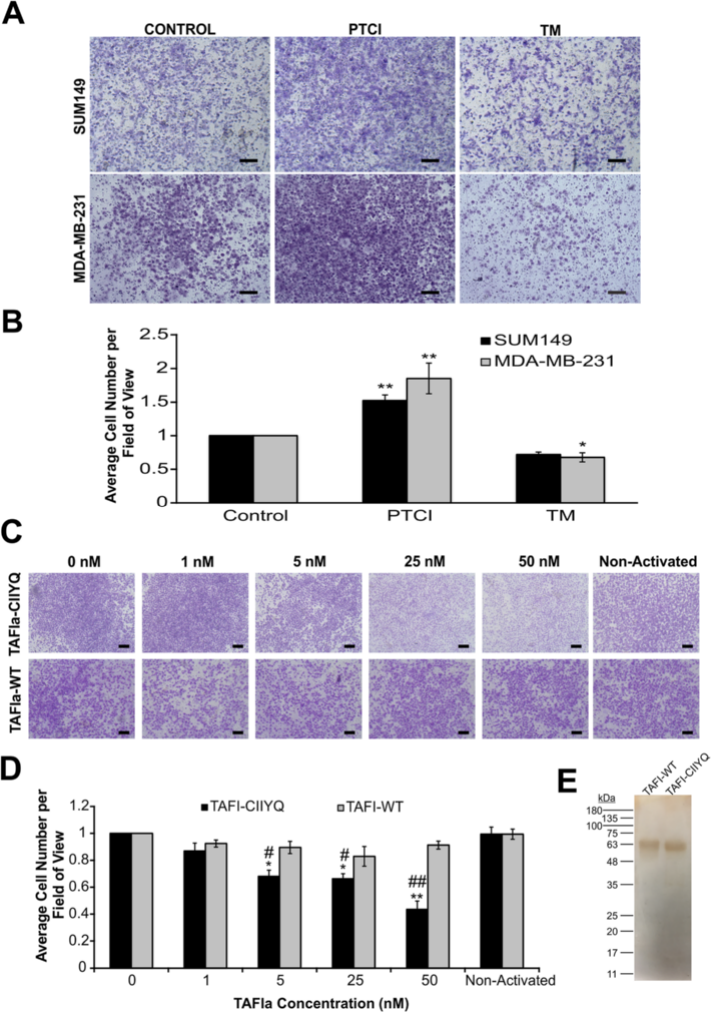
The effects of PTCI, TM and TAFIa on breast cancer cell invasion. **a** SUM149 and MDA-MB-231 cells were subjected to invasion assays in the presence of 10 μg/mL PTCI or 10 nM TM or in the absence of these additions (Control). The number of invaded cells were imaged and counted in five fields of view. Images shown were taken at 4× magnification. Scale bars: 200 μm. **b** Quantification of invaded cells, relative to control (absence of treatment). The data represent the average cell number per field of view ± SEM from at least 4 independent experiments. *: *p* <0.05 and **: *p* <0.01 relative to control. **c** Cells were treated with varying concentrations (1–50 nM) of either TAFIa-WT or TAFIa-CIIYQ or 50 nM mock-activated TAFI-WT or TAFI-CIIYQ (Non-Activated) for 24 h. Invasion was imaged as in (**a**). **d** Quantification of invaded cells, relative to control (absence of TAFIa). The data represent the average cell number per field of view ± SEM from at least 3 independent experiments. *: *p* <0.05 and **: *p* <0.01 relative to control; #: *p* <0.05 and ## *p* <0.01 relative to TAFIa-WT. **e** To demonstrate the purity of the recombinant TAFI proteins, TAFI-WT and TAFI-CIIYQ (500 ng each) was subjected to SDS-PAGE on a 12 % polyacrylamide gel followed by staining with silver. The positions of migration of molecular weight markers are shown to the left of the gel

**Fig. 4:**
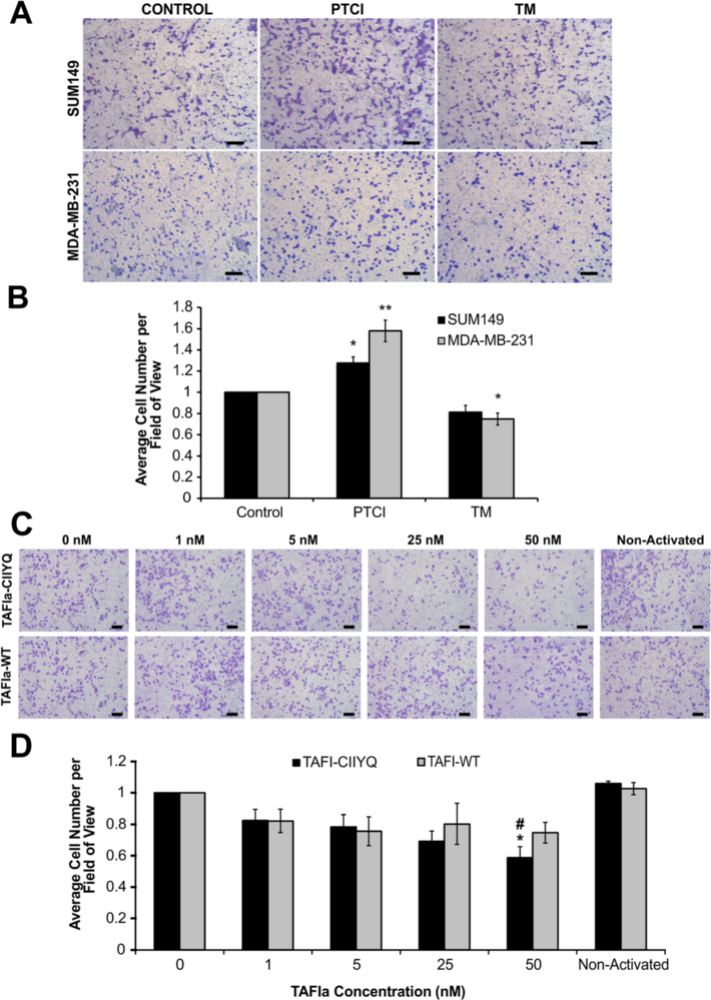
The effects of PTCI, TM and TAFIa on breast cancer cell migration. **a** SUM149 and MDA-MB-231 cells were subjected to migration assays in the presence of 10 μg/mL PTCI or 10 nM TM or in the absence of these additions (Control). The number of migrated cells were imaged and counted in five fields of view. Images shown were taken at 4× magnification. Scale bars: 200 μm. **b** Quantification of migrated cells, relative to control (absence of treatment). The data represent the average cell number per field of view ± SEM from at least 4 independent experiments. *: *p* <0.05 and **: *p* <0.01 relative to control. **c** Cells were treated with varying concentrations of either TAFIa-WT or TAFIa-CIIYQ (1–50 nM) for 24 h. Cells were also treated with 50 nM mock-activated TAFI-WT or TAFI-CIIYQ (Non-Activated). Migration was imaged as in (**a**). **d** Quantification of migrated cells, relative to control in the absence of TAFIa. The data represent the average cell number per field of view ± SEM from at least three independent experiments. *: *p* <0.05 relative to control; #: *p* <0.05 relative to TAFIa-WT

We examined the effect of TAFIa on cell invasion of MDA-MB-231 and SUM149 cell lines by inhibition of TAFIa using the specific competitive inhibitor PTCI. Both cell lines should be sensitive to the effects of PTCI as they both express TM and therefore presumably have the capacity to support TAFI activation. Inhibition of TAFIa using 10 μg/mL PTCI resulted in a significant increase in invasion (Fig. 3a, b) and migration (Fig. 4a, b) of both MDA-MB-231 and SUM149 cells. In addition, treatment with 10 nM of the cofactor TM resulted in an approximately 30 % decrease in invasion in both SUM149 and MDA-MB-231 cell lines (Fig. 3a, b) as well as decreases in migration of MDA-MB-231 and SUM149 cells by 30 and 20 %, respectively (Fig. 4a, b). These results indicate that modulation of TAFI activity can affect invasion and migration potential of breast cancer cells.

To directly assess the effect of TAFIa on cell invasion we used wild-type TAFI (TAFI-WT) as well as a stable variant (TAFI-CIIYQ) (Fig. 3c, d). While treatment with activated TAFI-WT (TAFIa-WT) did not affect cell invasion, we observed a dose-dependent decrease in MDA-MB-231 cell invasion upon treatment with TAFIa-CIIYQ. The highest dose of TAFIa-CIIYQ (50 nM) resulted in an approximately 60 % decrease in invasion of MDA-MB-231 cells (Fig. 3c, d). Notably, this effect was only observed with the TAFIa enzyme and not the (non-activated) TAFI zymogen. In addition, we assessed the effect of TAFI and TAFIa on cell migration. We observed a dose-dependent decrease in cell migration of MDA-MB-231 cells when treated with either TAFIa-WT or TAFIa-CIIYQ (Fig. 4c, d), although significance was only reached at 50 nM TAFIa-CIIYQ. Treatment with the non-activated TAFI did not have an effect on migration, as we observed with cell invasion. These results demonstrate directly that TAFIa can inhibit breast cancer cell invasion and migration in vitro.

### TAFIa does not affect breast cancer cell proliferation

**Fig. 5:**
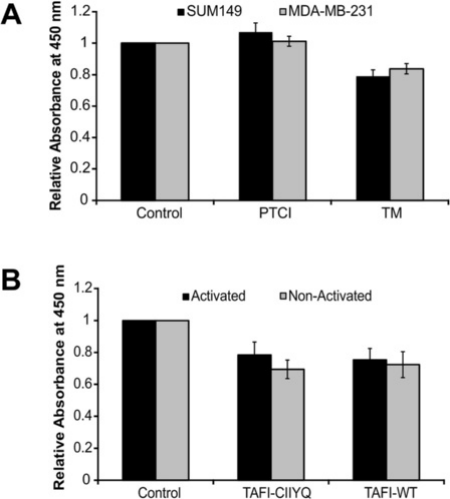
The effects of PTCI and TM on cell proliferation of breast cancer cell lines. MDA-MB-231 and SUM149 cells were subjected to the WST-1 assay as an index of proliferation. **a** Cells were treated with 10 μg/mL PTCI or 10 nM TM, for 24 h. **b** MDA-MB-231 cells were treated with 50 nM of the indicated TAFI variants, either with (Activated) or without (Non-Activated) prior treatment with thrombin-TM. Following the treatment, the WST-1 assay was conducted and metabolic activity of the cells assessed by measuring absorbance at 450 nm. The data represent the average cell number per field of view ± SEM from at least three independent experiments and are relative to the control in the absence of treatment

We next wanted to determine whether manipulation of TAFI impacted cell proliferation. Treatment with 10 μg/mL PTCI had no effect while treatment with 10 nM TM slightly decreased cell proliferation (as measured by a metabolic assay), albeit to an extent that was not statistically significant (Fig. 5). This result is in keeping with previous study conducted in melanoma cells [30]. As shown in Fig. 5b, treatment of MDA-MB-231 cells with both variants of TAFI resulted in a slight, albeit again non-significant, decrease in cell proliferation. This result was independent of TAFI activation, as addition of TAFIa gave the same result as addition of the zymogen.

### TAFIa inhibits DQ-collagen type IV proteolysis by MDA-MB-231 and SUM149 cells

**Fig. 6:**
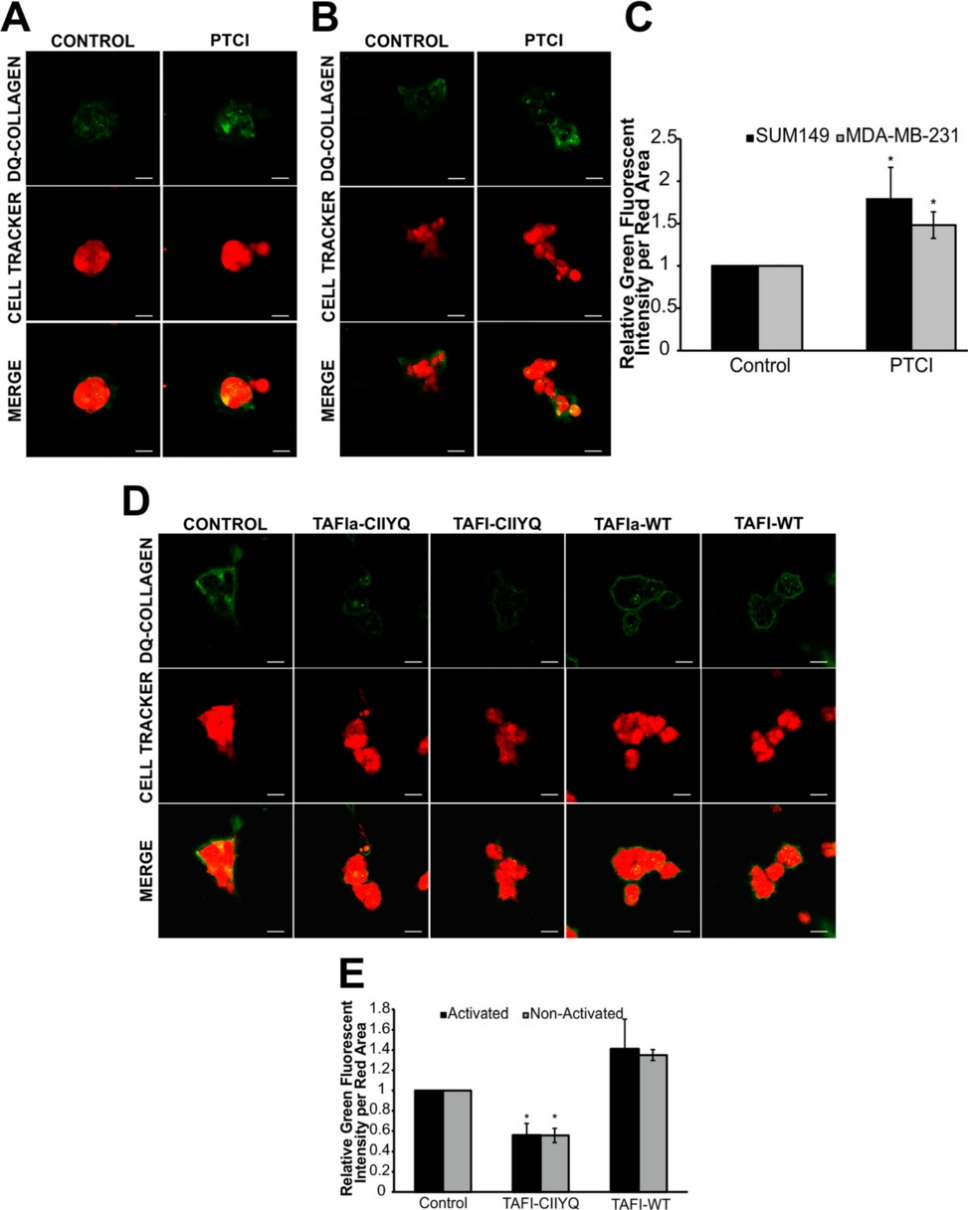
Inhibition of TAFIa increases proteolysis of DQ-collagen type IV by breast cancer cells. SUM149 (**a**) and MDA-MB-231 (**b**) cells labeled with CellTracker *Orange* (shown in *red*) were grown on reconstituted basement membrane extract containing 25 μg/mL DQ-collagen IV. Cells were treated with 10 μg/mL PTCI for 48 h. Confocal images were taken following the 48 h treatment. DQ-collagen degradation products were observed in *green*. Scale bars: 20 μm. **c** DQ-collagen fluorescence was quantified and normalized to the area of CellTracker *Orange* fluorescence. The data are represented as the *green* fluorescent intensity per area of the *red* fluorescence, averaged across the entire z-stack and expressed relative to untreated cells (Control). The data represent the mean ± SEM of three independent experiments, each carried out in triplicate. *: *p* <0.05 versus Control. **d** MDA-MB-231 cells labeled with CellTracker Orange (shown in *red*) were grown on rBM containing 25 μg/mL DQ-collagen IV and treated with 50 nM of either TAFIa-WT or TAFIa-CIIYQ, or with 50 nM mock-activated TAFI-WT or TAFI-CIIYQ. Confocal images of cells (*red*) and DQ-collagen IV degradation products (*green*) were captured after 48 h. Scale bars: 20 μm. **e** Degradation of DQ-collagen IV was quantified as in (**c**). The data represent the mean ± SEM of three independent experiments, each carried out in triplicate. *: *p* <0.05 versus Control

We examined the effect of TAFIa on degradation of DQ-collagen type IV by MDA-MB-231 and SUM149 cells. Inhibition of TAFIa using 10 μg/mL PTCI resulted in a significant increase in collagen IV proteolysis by both MDA-MB-231 and SUM149 cells (Fig. 6a-c). DQ-collagen degradation products were localized both intracellularly (represented by the yellow in the merged images) and extracellularly.

Next, we sought to directly investigate the effect of TAFIa on DQ-collagen type IV degradation. MDA-MB-231 cells were treated with either activated or mock-activated TAFI variants, TAFI-WT or TAFI-CIIYQ (Fig. 6d). Intriguingly, addition of TAFI-CIIYQ resulted in a decrease in collagen type IV proteolysis of approximately 30 %, irrespective of whether this variant was activated or not (Fig. 6e). Addition of wild-type TAFI or TAFIa had no significant effect on proteolysis.

Taken together, these results demonstrate a role for TAFIa in preventing collagen degradation.

## Discussion

TM has been shown to be an anti-metastatic factor in animal studies [19, 31] and low levels of tumor TM have been shown to be a negative prognostic indicator in breast cancer [20]. The anti-metastatic function of TM has been shown to reside in its ability to bind to thrombin [19]. We report here that a molecular target of the thrombin TM complex – namely, TAFI – can inhibit pro-metastatic behaviours of breast cancer cells in vitro including invasion, migration, and extracellular proteolysis.

The thrombin-TM complex activates TAFI over 1000-fold more efficiently than thrombin alone, and 20-fold more efficiently than plasmin [32]. Accordingly, we would hypothesize thrombin-TM to be a relevant activator of TAFI in the tumor microenvironment, particularly in the absence of a strong thrombogenic stimulus that would result in generation of large amounts of thrombin and plasmin. We found that the more malignant cell lines tended to express less mRNA encoding TM (Fig. 1), and hence these cells would be less able to support TAFI activation. In other words, the more malignant cell lines are characterized by a relatively low capacity to generate potentially anti-metastatic TAFIa. Indeed, although Reijerkerk and coworkers found that absence of TAFI did not affect the growth and metastasis of B16 melanoma or Lewis lung carcinoma cell lines in mice, these cell lines are unable to support TAFI activation [33].

Although we were able to detect expression of mRNA encoding TAFI in breast cancer cells, there was no apparent correlation with malignancy (Fig. 1). This observation evokes the concept that the limiting factor in the anti-metastatic TAFI pathway may not be TAFI itself, but instead the presence of TM that is required for TAFI activation in this milieu. Indeed, several studies have shown that plasma TAFI concentrations are elevated in breast cancer patients and are positively correlated with endpoints such as poor prognosis and risk of recurrence [34–36]. These findings are the opposite of what would be expected given the ability of TAFIa to inhibit pro-metastatic behaviours such as invasion that we have documented in this study. Moreover, a common single nucleotide polymorphism in *CPB2* encoding a more stable TAFIa species was also more frequent in breast cancer patients than controls [35, 36]. In the breast cancer microenvironment, TM may be provided by the breast cancer cells and/or by stromal cells such as endothelial cells and macrophages [37]. TAFI may arise from tumor cells or macrophages, but more likely from blood plasma present due to leaky tumor microvasculature [38].

We showed that TAFI can inhibit plasminogen activation on the breast cancer cell surface (Fig. 2). Carboxyl-terminal lysine binding sites on cell surface receptors play a crucial role in plasminogen activation [39]. TAFIa cleaves carboxyl-terminal lysine residues from various protein and peptide substrates. Plasmin has a series of pro-metastatic effects including direct proteolysis of ECM and basement membrane proteins, activation of latent pro-MMPs, and liberation of latent growth factors from the extracellular matrix [40]. The decrease in plasmin generation in the presence of TAFIa likely results in decreased activation of latent MMPs in the extracellular milieu, amplifying the effect of TAFIa. It is notable that inhibition of TAFIa had a greater effect on cell invasion than cell migration (Figs. 3 and 4). The former requires cells to degrade the matrix while the latter does not, and is an index merely of how fast cells can move. While the PAS has been shown to be important for cell movement [40], our data suggest a key role for TAFI in blocking proteolysis of collagen and other extracellular matrix components.

TAFIa activity is regulated by the intrinsic instability of the enzyme. While the TAFI zymogen is stable, the enzyme undergoes a spontaneous conformational change that causes loss of activity [21, 41]. The half-life of TAFIa at body temperature is 8–15 min [42]. When we added purified recombinant TAFIa to our invasion assays, we saw no effect (Fig. 3). This is not surprising as the invasion assay is conducted over 24 h and most of the TAFIa activity would have disappeared within the first hour. Indeed, when we used a stable TAFIa variant (TAFIa-CIIYQ) with a much longer half-life (1140 min [43]) we did observe a dose-dependent decrease in cell invasion (Fig. 3). Addition of the non-activated forms of wild-type TAFI or TAFI-CIIYQ had no effect on invasion (Fig. 3), indicating that TAFI zymogen concentration was not a limiting factor under these conditions. However, in cell migration assays, both enzymes appeared to have an effect (Fig. 4), although the decreases caused by wild-type TAFIa were non-significant. Again, addition of the zymogens had no effect (Fig. 4). The migration assays may have been influenced at an early stage by the presence of TAFIa, for example if most of the migration occurred in the first hours of the 24-hour incubation. Assays of the metabolic activity of the breast cancer cell lines showed minimal impact of manipulation of TAFIa activity (Fig. 5). These findings suggest that the effect of TAFIa on invasion and migration can not be attributed to changes in cell proliferation.

Inhibition of TAFIa enhanced DQ-collagen degradation, indicating an important role for TAFIa in mediating collagen proteolysis (Fig. 6). Unlike cell migration, in the proteolysis assays both the wild-type and mutant zymogens of TAFI along with their active counterparts resulted in a decrease in collagen degradation (Fig. 6). The 48-hour incubation for the proteolysis assays may have allowed for ongoing activation of the added TAFI to have manifested in less accumulation of degraded DQ-collagen type IV. In the breast tumor microenvironment, we would expect continuous activation of TAFI through the action on small amounts of thrombin generated *in situ* interacting with TM on the surface of different cell types in this environment. We observed the presence of DQ-collagen type IV proteolysis both extracellularly and intracellularly (Fig. 6). The proteases and pathways involved in pericellular proteolysis versus intracellular proteolysis are distinct [44, 45]. Pericellular proteolysis involves MMPs, serine proteases (including components of the plasminogen activation system), and cysteine proteases [44, 45]. Intracellular proteolysis involves endocytosis of DQ-collagen IV by the live cells followed by degradation of the substrate in the lysosome [45–47]. Intracellular proteolysis can also occur after partial degradation of DQ-collagen type IV by pericellular proteases with subsequent endocytosis and further degradation in the lysosome [48]. As such, an increase in intracellular proteolysis also reflects the extent of pericellular proteolysis. We saw effects of manipulation of TAFIa activity on both pericellular and intracellular proteolysis (Fig. 6), but since TAFIa acts outside the cell, we would argue that TAFIa directly affects pericellular proteolysis, with an indirect effect on intracellular proteolysis.

## Conclusions

In conclusion, we have demonstrated for the first time that activation of TAFI results in a suppression of breast cancer cell invasion and migration. Our data are consistent with the idea that TAFIa mediates these effects by inhibiting pericellular plasminogen activation and by attenuating proteolysis of collagen type IV. Further work will be required to define the full spectrum of the effects of TAFIa on specific proteases and protease targets in the breast tumour microenvironment. Our results indicate that promotion of TAFI activation in this microenvironment, either by provision of soluble TM or stabilization of TAFIa, would have anti-metastatic effects.

## Abbreviations

ECM: extracellular matrix
FDPs: fibrin degradation products
MMP: matrix metalloproteinase
PAS: plasminogen activation system
PTCI: potato tuber carboxypeptidase inhibitor
TAFI: thrombin activatable fibrinolysis inhibitor
TAFIa: activated thrombin activatable fibrinolysis inhibitor
TM: thrombomodulin
tPA: tissue-type plasminogen activator
uPA: urokinase plasminogen activator
uPAR: urokinase plasminogen activator receptor

## References

[1] Hanahan D, Weinberg RA (2011). Hallmarks of cancer: the next generation. Cell.

[2] Criscitiello C, Esposito A, Curigliano G (2014). Tumor-stroma crosstalk: targeting stroma in breast cancer. Curr Opin Oncol.

[3] Lu P, Weaver VM, Werb Z (2012). The extracellular matrix: a dynamic niche in cancer progression. J Cell Biol.

[4] Dano K, Behrendt N, Hoyer-Hansen G, Johnsen M, Lund LR, Ploug M, Romer J (2005). Plasminogen activation and cancer. Thromb Haemost.

[5] Wong MS, Sidik SM, Mahmud R, Stanslas J (2013). Molecular targets in the discovery and development of novel antimetastatic agents: current progress and future prospects. Clin Exp Pharmacol Physiol.

[6] Carmeliet P, Collen D (1998). Development and disease in proteinase-deficient mice: role of the plasminogen, matrix metalloproteinase and coagulation system. Thromb Res.

[7] Deryugina EI, Quigley JP (2012). Cell surface remodeling by plasmin: a new function for an old enzyme. J Biomed Biotechnol.

[8] Binder BR, Mihaly J, Prager GW (2007). uPAR-uPA-PAI-1 interactions and signaling: a vascular biologist’s view. Thromb Haemost.

[9] Lund IK, Illemann M, Thurison T, Christensen IJ, Hoyer-Hansen G (2011). uPAR as anti-cancer target: evaluation of biomarker potential, histological localization, and antibody-based therapy. Curr Drug Targets.

[10] Bertina RM, van Tilburg NH, Haverkate F, Bouma BN, von dem Borne PA, Meijers JC, Campbell W, Eaton D, Hendriks DF, Willemse JL (2006). Discovery of thrombin activatable fibrinolysis inhibitor (TAFI). J Thromb Haemost.

[11] Lin JH, Garand M, Zagorac B, Schadinger SL, Scipione C, Koschinsky ML, Boffa MB (2011). Identification of human thrombin-activatable fibrinolysis inhibitor in vascular and inflammatory cells. Thromb Haemost.

[12] Campbell WD, Lazoura E, Okada N, Okada H (2002). Inactivation of C3a and C5a octapeptides by carboxypeptidase R and carboxypeptidase N. Microbiol Immunol.

[13] Myles T, Nishimura T, Yun TH, Nagashima M, Morser J, Patterson AJ, Pearl RG, Leung LL (2003). Thrombin activatable fibrinolysis inhibitor, a potential regulator of vascular inflammation. J Biol Chem.

[14] Redlitz A, Tan AK, Eaton DL, Plow EF (1995). Plasma carboxypeptidases as regulators of the plasminogen system. J Clin Invest.

[15] Swaisgood CM, Schmitt D, Eaton D, Plow EF (2002). In vivo regulation of plasminogen function by plasma carboxypeptidase B. J Clin Invest.

[16] Boffa MB, Koschinsky ML (2007). Curiouser and curiouser: recent advances in measurement of thrombin-activatable fibrinolysis inhibitor (TAFI) and in understanding its molecular genetics, gene regulation, and biological roles. Clin Biochem.

[17] Ceresa E, De Maeyer M, Jonckheer A, Peeters M, Engelborghs Y, Declerck PJ, Gils A (2007). Comparative evaluation of stable TAFIa variants: importance of alpha-helix 9 and beta-sheet 11 for TAFIa (in)stability. J Thromb Haemost.

[18] Higuchi T, Nakamura T, Kakutani H, Ishii H (2009). Thrombomodulin suppresses invasiveness of HT1080 tumor cells by reducing plasminogen activation on the cell surface through activation of thrombin-activatable fibrinolysis inhibitor. Biol Pharm Bull.

[19] Horowitz NA, Blevins EA, Miller WM, Perry AR, Talmage KE, Mullins ES, Flick MJ, Queiroz KC, Shi K, Spek CA (2011). Thrombomodulin is a determinant of metastasis through a mechanism linked to the thrombin binding domain but not the lectin-like domain. Blood.

[20] Kim SJ, Shiba E, Ishii H, Inoue T, Taguchi T, Tanji Y, Kimoto Y, Izukura M, Takai S (1997). Thrombomodulin is a new biological and prognostic marker for breast cancer: an immunohistochemical study. Anticancer Res.

[21] Boffa MB, Wang W, Bajzar L, Nesheim ME (1998). Plasma and recombinant thrombin-activable fibrinolysis inhibitor (TAFI) and activated TAFI compared with respect to glycosylation, thrombin/thrombomodulin-dependent activation, thermal stability, and enzymatic properties. J Biol Chem.

[22] Romagnuolo R, Marcovina SM, Boffa MB, Koschinsky ML (2014). Inhibition of plasminogen activation by apo(a): role of carboxyl-terminal lysines and identification of inhibitory domains in apo(a). J Lipid Res.

[23] Deutsch DG, Mertz ET (1970). Plasminogen: purification from human plasma by affinity chromatography. Science.

[24] Victor BC, Anbalagan A, Mohamed MM, Sloane BF, Cavallo-Medved D (2011). Inhibition of cathepsin B activity attenuates extracellular matrix degradation and inflammatory breast cancer invasion. Breast Cancer Res.

[25] Otsu N (1975). A threshold selection method from gray-level histograms. Automatica.

[26] Nagaraja GM, Othman M, Fox BP, Alsaber R, Pellegrino CM, Zeng Y, Khanna R, Tamburini P, Swaroop A, Kandpal RP (2006). Gene expression signatures and biomarkers of noninvasive and invasive breast cancer cells: comprehensive profiles by representational difference analysis, microarrays and proteomics. Oncogene.

[27] Liu PL, Tsai JR, Chiu CC, Hwang JJ, Chou SH, Wang CK, Wu SJ, Chen YL, Chen WC, Chen YH (2010). Decreased expression of thrombomodulin is correlated with tumor cell invasiveness and poor prognosis in nonsmall cell lung cancer. Mol Carcinog.

[28] Menschikowski M, Hagelgans A, Tiebel O, Vogel M, Eisenhofer G, Siegert G (2012). Regulation of thrombomodulin expression in prostate cancer cells. Cancer Lett.

[29] Hanly AM, Hayanga A, Winter DC, Bouchier-Hayes DJ (2005). Thrombomodulin: tumour biology and prognostic implications. Eur J Surg Oncol.

[30] Zhang Y, Weiler-Guettler H, Chen J, Wilhelm O, Deng Y, Qiu F, Nakagawa K, Klevesath M, Wilhelm S, Bohrer H (1998). Thrombomodulin modulates growth of tumor cells independent of its anticoagulant activity. J Clin Invest.

[31] Hosaka Y, Higuchi T, Tsumagari M, Ishii H (2000). Inhibition of invasion and experimental metastasis of murine melanoma cells by human soluble thrombomodulin. Cancer Lett.

[32] Miah MF, Boffa MB (2009). Functional analysis of mutant variants of thrombin-activatable fibrinolysis inhibitor resistant to activation by thrombin or plasmin. J Thromb Haemost.

[33] Reijerkerk A, Meijers JC, Havik SR, Bouma BN, Voest EE, Gebbink MF (2004). Tumor growth and metastasis are not affected in thrombin-activatable fibrinolysis inhibitor-deficient mice. J Thromb Haemost.

[34] Kaftan O, Kasapoglu B, Koroglu M, Kosar A, Yalcin SK (2011). Thrombin-activatable fibrinolysis inhibitor in breast cancer patients. Med Princ Pract.

[35] Chengwei X, Xiaoli M, Yuan Z, Li P, Shengjiang W, Chao Y, Yunshan W (2013). Plasma thrombin-activatable fibrinolysis inhibitor levels and its Thr325Ile polymorphism in breast cancer. Blood Coagul Fibrinolysis.

[36] Fawzy MS, Mohammed EA, Ahmed AS, Fakhr-Eldeen A (2015). Thrombin-activatable fibrinolysis inhibitor Thr325Ile polymorphism and plasma level in breast cancer: A pilot study. Meta Gene.

[37] Conway EM (2012). Thrombomodulin and its role in inflammation. Semin Immunopathol.

[38] Fukumura D, Jain RK (2007). Tumor microvasculature and microenvironment: targets for anti-angiogenesis and normalization. Microvasc Res.

[39] Godier A, Hunt BJ (2013). Plasminogen receptors and their role in the pathogenesis of inflammatory, autoimmune and malignant disease. J Thromb Haemost.

[40] Mekkawy AH, Pourgholami MH, Morris DL (2014). Involvement of urokinase-type plasminogen activator system in cancer: an overview. Med Res Rev.

[41] Marx PF, Brondijk TH, Plug T, Romijn RA, Hemrika W, Meijers JC, Huizinga EG (2008). Crystal structures of TAFI elucidate the inactivation mechanism of activated TAFI: a novel mechanism for enzyme autoregulation. Blood.

[42] Schneider M, Boffa M, Stewart R, Rahman M, Koschinsky M, Nesheim M (2002). Two naturally occurring variants of TAFI (Thr-325 and Ile-325) differ substantially with respect to thermal stability and antifibrinolytic activity of the enzyme. J Biol Chem.

[43] Ceresa E, Peeters M, Declerck PJ, Gils A (2007). Announcing a TAFIa mutant with a 180-fold increased half-life and concomitantly a strongly increased antifibrinolytic potential. J Thromb Haemost.

[44] Sameni M, Dosescu J, Moin K, Sloane BF (2003). Functional imaging of proteolysis: stromal and inflammatory cells increase tumor proteolysis. Mol Imaging.

[45] Sameni M, Moin K, Sloane BF (2000). Imaging proteolysis by living human breast cancer cells. Neoplasia.

[46] Ahram M, Sameni M, Qiu RG, Linebaugh B, Kirn D, Sloane BF (2000). Rac1-induced endocytosis is associated with intracellular proteolysis during migration through a three-dimensional matrix. Exp Cell Res.

[47] Sameni M, Dosescu J, Sloane BF (2001). Imaging proteolysis by living human glioma cells. Biol Chem.

[48] Everts V, van der Zee E, Creemers L, Beertsen W (1996). Phagocytosis and intracellular digestion of collagen, its role in turnover and remodelling. Histochem J.

